# Carotid Artery Longitudinal Displacement, Cardiovascular Disease and Risk Factors: The Multi-Ethnic Study of Atherosclerosis

**DOI:** 10.1371/journal.pone.0142138

**Published:** 2015-11-06

**Authors:** Adam D. Gepner, Laura A. Colangelo, Nicole Reilly, Claudia E. Korcarz, Joel D. Kaufman, James H. Stein

**Affiliations:** 1 Division of Cardiovascular Medicine, Department of Medicine, University of Wisconsin School of Medicine and Public Health, Madison, WI, United States of America; 2 Department of Preventive Medicine, Northwestern University Feinberg School of Medicine, Chicago, IL, United States of America; 3 Department of Epidemiology, University of Washington School of Public Health, Seattle, Washington, United States of America; Temple University School of Medicine, UNITED STATES

## Abstract

**Background:**

Associations between carotid artery longitudinal displacement, cardiovascular disease risk factors, and events were evaluated in a large, multi-ethnic cohort.

**Materials and Methods:**

A novel, reproducible protocol was developed for measuring right common carotid artery longitudinal displacement using ultrasound speckle-tracking. Total longitudinal displacement was measured in 389 randomly selected participants from the Multi-Ethnic Study of Atherosclerosis that were free of cardiovascular disease at baseline. Univariate analyses and Pearson Correlations were used to define relationships between longitudinal displacement with traditional cardiovascular risk factors and traditional measures of arterial stiffness. Hazard ratios of longitudinal displacement for cardiovascular disease and coronary heart disease events were compared using Cox proportional hazards models.

**Results:**

Participants were a mean (standard deviation) 59.0 (8.7) years old, 48% female, 39% White, 26% Black, 22% Hispanic, and 14% Chinese. They had 19 (4.9%) cardiovascular disease and 14 (3.6%) coronary heart disease events over a mean 9.5 years of follow-up. Less longitudinal displacement was associated with Chinese (β = -0.11, p = 0.02) compared to White race/ethnicity and greater longitudinal displacement was associated with higher carotid intima-media thickness (β = 0.26, p = 0.004). Longitudinal displacement was not associated with other cardiovascular disease risk factors or markers of arterial stiffness. After adjustment for age and sex, and heart rate, Chinese race/ethnicity (β = -0.10, p = 0.04) and carotid intima-media thickness (β = 0.30 p = 0.003) were associated independently with longitudinal displacement. Longitudinal displacement predicted coronary heart disease (Hazard ratio [HR] 3.3, 95% Confidence intervals [CI] 0.96–11.14, p = 0.06) and cardiovascular disease (HR 2.1, 95% CI 0.6–7.3, p = 0.23) events.

**Conclusions:**

Less longitudinal displacement is associated with Chinese ethnicity and greater carotid artery longitudinal displacement is associated with thicker intima-media thickness. Longitudinal displacement may predict adverse coronary heart disease and cardiovascular disease events.

## Introduction

Arterial stiffness is associated with development of hypertension, heart failure, stroke, and myocardial infarction [1, 2]. The most commonly obtained carotid arterial stiffness measurements, distensibility coefficient (DC) and Young’s Elastic Modulus (YEM), evaluate radial or circumferential arterial displacement [2, 3]. Because they assume that longitudinal arterial movement is negligible compared to radial movement, they are imperfect markers of arterial pathophysiology [4]. This assumption appears to be untrue, challenging the validity of research studies that used traditional measures of local arterial stiffness and stimulating interest in characteristics of longitudinal displacement (LD) [4–6]. Because of recent advances in imaging technology, longitudinal movements (along the long axis of the vessel) of the arterial wall can be measured non-invasively using ultrasound [7–10].

Velocity vector imaging (VVI) is a non-invasive ultrasound technique that uses a two-dimensional (2D) speckle tracking algorithm. In grey scale images, backscattered ultrasound signals from adjacent structures cause a random “speckle” pattern, such that each small image region has a unique pattern of acoustic markers or speckles. VVI uses a pattern-matching algorithm to accurately track these speckles and analyze motion and can be used to measure displacement (mm) [7, 8, 11]. Measurements of carotid artery LD and their associations with CVD risk factors are not clear, especially in patients without known CVD. Prior studies that used carotid artery VVI to describe arterial motion were small [8, 9, 12] or did not evaluate longitudinal motion [13–15]. Those studies that did evaluate longitudinal movement had conflicting results with unclear risk factor associations or made geometric assumptions about arterial movements that may not be correct [7, 8, 16, 17].

MESA provides a unique opportunity to study carotid arterial LD in a large, multi-ethnic cohort, initially free of cardiovascular disease (CVD), and to compare these measurements with traditional measures of arterial stiffness such as Young’s Elastic Modulus (YEM) and Distensibility Coefficient (DC). A standardized protocol was developed to assess LD of the carotid artery using ultrasound and it was hypothesized that carotid artery LD would be associated with traditional CVD risk factors and future coronary heart disease (CHD) and CVD events.

## Materials and Methods

### Study Participants and Design

The Multi-Ethnic Study of Atherosclerosis (MESA) is a large prospective, cohort study investigating the prevalence, causes, and progression of subclinical CVD. MESA is a population-based sample of 6,814 men and women aged 45 to 84 years, free of known CVD at baseline, recruited from 6 United States communities (Baltimore, Maryland; Chicago, Illinois; Forsyth County, North Carolina; Los Angeles County, California; Northern Manhattan, New York; and St. Paul, Minnesota). The study objectives and design have been published previously. All subjects provided written informed consent. This study was approved by the University of Wisconsin Institutional Review Board. These analyses were from a randomly selected subset of 500 MESA participants with Exam 1 images of the right common carotid artery. Of these images, 65 (13%) were excluded from the analysis because they did not have YEM and DC measurements. An additional 46 (9%) subjects were excluded from the LD analysis due to probe movement or respiratory translation of the carotid artery, leaving 389 participants for the final analyses.

Demographic, medical history and laboratory data for the present study were obtained from the first (July 2000 to August 2002) examination of the MESA cohort. Hypertension was defined as SBP ≥140 mmHg, diastolic blood pressure ≥90 mmHg, or the use of antihypertensive medications. Diabetes mellitus was defined as fasting blood glucose ≥126 mg/dL or the use of antiglycemic medications. Impaired fasting glucose was defined as blood glucose from ≥100 but <126 mg/dL. Total and high-density lipoprotein cholesterol levels were measured from blood samples obtained after a 12-hour fast. Low-density lipoprotein cholesterol was calculated using the Friedewald equation [18].

### B-mode Ultrasound and Brachial Blood Pressure Measurements

At exam 1, B-mode ultrasound video loop recordings of a longitudinal section of the distal right common carotid artery were recorded on videotape using a Logiq 700 ultrasound system (General Electric Medical Systems, transducer frequency 13 MHz). Video images were digitized using a Medical Digital Recording (MDR) device (PACSGEAR, Pleasanton, CA) and converted into DICOM compatible digital records [19, 20]. Brachial artery blood pressures were obtained using a standardized protocol with an automated upper arm sphygmomanometer (DINAMAP, GE Medical Systems, Milwaukee, WI) after resting in the supine position for 10 minutes. Ultrasound images were reviewed and interpreted by the MESA Air Carotid Ultrasound Reading Center (the University of Wisconsin Atherosclerosis Imaging Research Program, Madison, WI).

### Measurement of Longitudinal Displacement

**Fig 1:**
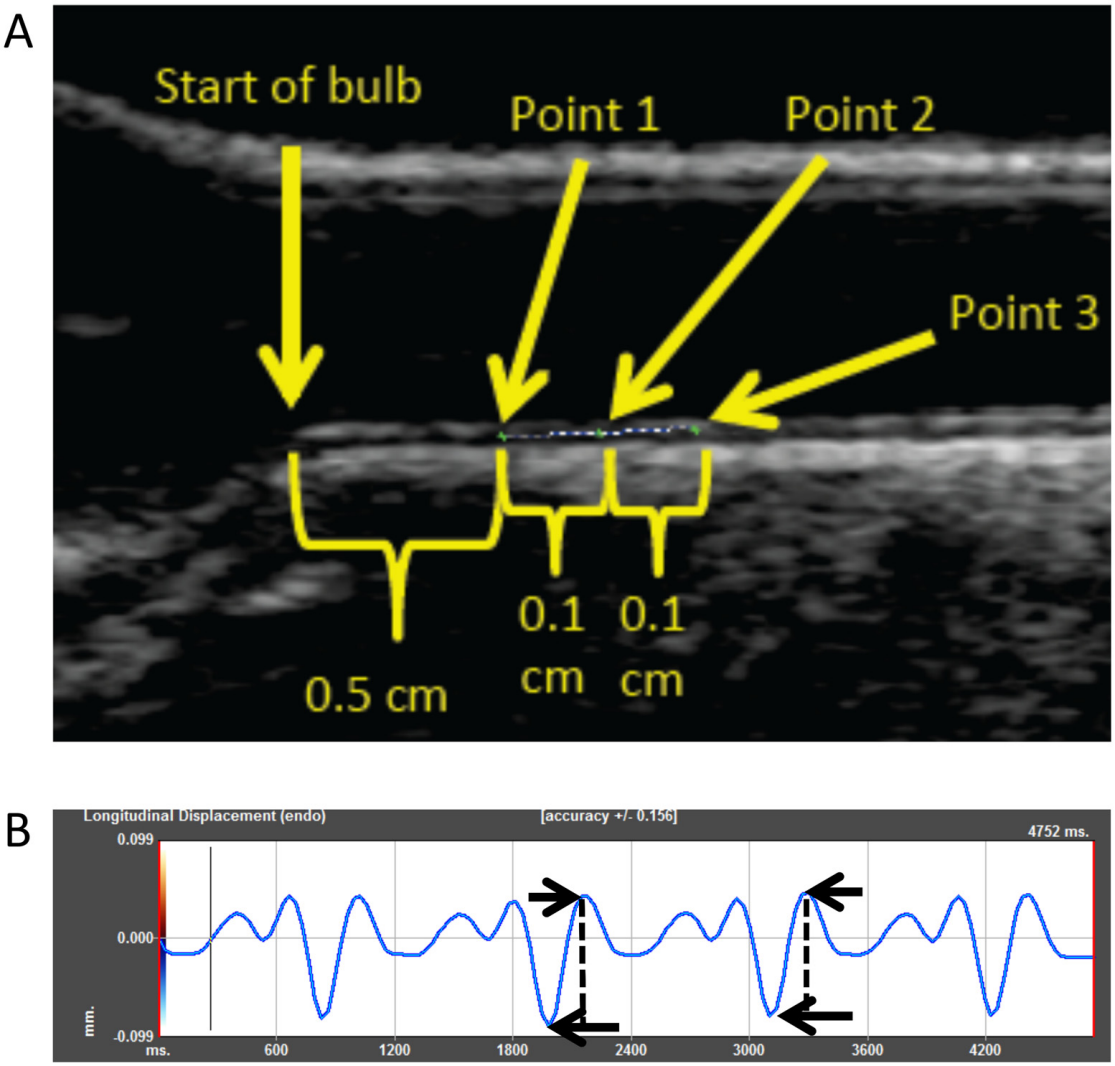
Measurement of Carotid Artery Longitudinal Displacement. **Panel A.** Image of the right common carotid artery. The overlay schematic of the tracing point locations (green circles) are used for the determination of longitudinal displacement. A region of interest was identified by measuring 0.5 cm caudal from the carotid bulb along the far wall of the distal common carotid artery, using an onscreen measuring tool (Microsoft Windows Ruler). The second and third points were placed 0.1 cm apart, extending caudally. Each point that was placed at the intima-media interface is tracked using a “speckle tracking” algorithm (TomTec, Unterscheissheim, Germany) that tracks specific pixel movement during the cardiac cycle. **Panel B.** Sample output of a longitudinal displacement waveform indicated by the solid blue line. This was generated using a “speckle tracking” algorithm (TomTec, Unterscheissheim, Germany) with a free-trace feature that makes no geometric assumptions about the shape of the object being analyzed. Longitudinal displacement (mm) is on the y-axis and time (ms) is on the x-axis. Arrows mark the maximum and minimum displacement of the second and third beats in the ultrasound loop. The dotted lines represent the total longitudinal displacement for these beats. Reported longitudinal displacement values are averaged over two cardiac cycles.

A new technique and protocol was developed and validated for using VVI analysis software to measure carotid artery LD and velocity (TomTec, Unterscheissheim, Germany). One reader (ADG) performed all the VVI measurements using a free-trace feature to track the far wall of the right common carotid artery. A region of interest was identified by measuring 0.5 cm caudal from the carotid bulb along the far wall of the distal common carotid artery, using an onscreen measuring tool (Microsoft Windows Ruler). A second and third point were placed 0.1 cm apart extending caudally in the CCA (Fig 1, Panel A). All points were placed at the intima-media interface. The free-trace feature makes no geometric assumptions about the shape and movement of the object being analyzed. From the outputted waveform, LD was measured in 2 cardiac cycles (Fig 1, Panel B). The absolute value described the total LD of the carotid artery during the cardiac cycle. LD was averaged for the two beats. A subset of ultrasound loops from 25 participants were re-measured by a second reader (CEK) and assessed for inter-reader variability to ensure reproducibility of this new technique.

### Carotid Intima-Media Thickness and Traditional Arterial Stiffness Measurements

Ultrasound was also used to assess DC (10^−3^ mmHg^-1^), YEM (mmHg), and common carotid artery carotid intima-media thickness (IMT) [19, 20]. Mean and maximal IMT of the far wall of distal CCA (distal 1 cm, proximal to the carotid bifurcation point, where the distal CCA diameter remains uniform) and the proximal 1 cm of the ICA were measured in triplicate at the University of Wisconsin using a semi-automated border detection program (Syngo Arterial Health package, Siemens Medical Solutions, Malvern, PA) blinded to subject demographic and medical information. For DC and YEM, systolic and diastolic diameters were determined as the largest and smallest diameters during the cardiac cycle. All measurements were performed in triplicate from 2–3 consecutive cardiac cycles to derive mean internal diameter at peak systole and mean internal and external diameters at end-diastole using Access Point Web version 3.0 (Freeland Systems, LLC) [20]. Thoracic aortic distensibility was measured with magnetic resonance imaging [21]; small and large artery elasticity were measured using radial pulse contour analyses [21]. Each of these measurements was calculated using standard formulae and have been previously described in detail within MESA [19–22].

### Cardiovascular Disease Events

Participants were followed from the baseline examination through October 2011 for a mean of 9.5 years. They, or a proxy, were contacted by telephone every 9–12 months to inquire about interim hospital admissions, CVD outpatient diagnoses, and deaths. Events were verified with death certificates and medical records. Two physicians, blinded to study data, independently reviewed and classified CVD events. In cases of disagreement, a mortality and morbidity committee determined the final classification. Criteria for MESA CVD events have been published previously [23]. CVD was defined as CHD (definite or probable myocardial infarction, CHD death, resuscitated cardiac arrest, definite angina, and probable angina—if followed by coronary revascularization), stroke (fatal or non-fatal), or other atherosclerotic CVD death. A detailed description of the MESA follow-up methods is available at http://www.mesa-nhlbi.org.

### Statistical Analysis

Results are reported as mean (standard deviation) for continuous variables or percentages for categorical variables. Univariate analyses and Pearson Correlations were used to define relationships between LD with traditional CVD risk factors, traditional measures of arterial stiffness such as carotid artery DC, carotid artery YEM, aortic distensibility, and both small and large artery elasticity, carotid IMT, and socio-demographic factors. Multivariable regression models were used to evaluate independent predictors of LD. Sequential models were (1) unadjusted, (2) adjusted for age, race/ethnicity and gender, and (3) adjusted for cardiovascular risk factors, traditional stiffness measurements, and carotid IMT. Hazard ratios (HR) and 95% confidence intervals (CIs) of LD for CVD and CHD events were computed using Cox proportional hazards models with adjustment for age, sex and race. Statistical significance was set at p<0.05. All analyses were carried out with the use of SAS (Version 9.4, Cary, NC: SAS. Institute Inc.). Inter-reader variability was assessed with correlation coefficients and Bland-Altman plots.

## Results

### Baseline Characteristics

Baseline characteristics are presented in Table 1. Participants were a mean (standard deviation) 59.0 (8.7) years old and 187 (48.1%) were female. The participants selected were ethnically diverse; 38.6% were White, 26.0% Black, 21.9% Hispanic, and 13.6% were Chinese. Mean total LD during the cardiac cycle was 0.42 (0.31) mm. The mean YEM was 1526 (780) mmHg and the average DC was 3.2 (1.3) 10^−3^ mmHg^-1^. The mean carotid IMT was 0.835 (0.175) mm. The mean aortic distensibility was 2.0 (1.3) x 10^−3^ mmHg^-1^, small artery elasticity was 4.9 (3.1) mmHg, and large artery elasticity was 14.5 (5.3) mmHg.

**Table 1:** Participant Characteristics by Tertiles of Carotid Artery Longitudinal displacement.

	All Subjects (n = 389)	Tertile 1: (0.013–0.250) (n = 129)	Tertile 2: (0.252–0.456) (n = 131)	Tertile 3: (0.457–2.263) (n = 129)
Age (years)	59.0 (8.7)	59.2 (9.3)	58.6 (8.1)	59.0 (8.8)
Female sex (%)	187 (48.1)	51 (39.5)	68 (48.1)	68 (52.7)
Ethnicity (%)				
White	150 (38.6)	44 (34.1)	54 (41.2)	52 (40.3)
Black	101 (26.0)	35 (27.1)	35 (26.7)	31 (24.0)
Chinese	53 (13.6)	25 (19.4)	15 (11.5)	13 (10.1)
Hispanic	85 (21.9)	25 (19.4)	27 (20.6)	33 (25.6)
Blood pressure parameters				
Systolic blood pressure (mmHg)	123.2 (20.1)	125.3 (20.3)	121.1 (19.8)	123.2 (20.0)
Diastolic blood pressure (mmHg)	72.2 (10.1)	74.4 (10.6)	71.0 (9.2)	71.3 (10.1)
Pulse Pressure (mmHg)	51.0 (14.7)	50.9 (14.4)	50.1 (15.5)	51.8 (14.3)
Heart rate (bpm)	61.5 (8.7)	61.5 (8.9)	61.7 (9.1)	61.3 (8.3)
Hypertension (%)	149 (38.3)	53 (41.1)	44 (33.6)	52 (40.3)
Antihypertensive medications (%)	121 (31.1)	44 (34.1)	32 (24.4)	45 (34.9)
Diabetes mellitus status				
Impaired fasting glucose (N, %)	44 (11.3)	15 (11.7)	21 (16.0)	8 (6.2)
Untreated (N, %)	4 (1.0)	0 (0)	0 (0)	4 (3.1)
Treated (N, %)	28 (7.2)	8 (6.3)	9 (6.9)	11 (8.5)
Body mass index (kg/m^2^)	27.6 (5.0)	27.4 (4.7)	27.8 (5.2)	27.6 (5.1)
Waist Circumference(cm)	96.1 (13.0)	95.9 (12.8)	95.8 (13.0)	96.6 (13.1)
Former smoker (N, %)	137 (53.9)	51 (39.5)	35 (26.7)	51 (39.8)
Current smoker (N, %)	42 (10.8)	12 (9.3)	11 (8.4)	19 (14.8)
Small artery elasticity (mL/mmHg x 100)	4.96 (3.10)	4.95 (3.21)	4.82 (2.77)	5.12 (3.32)
Large artery elasticity (mL/mmHg x 100)	14.51 (5.28)	14.70 (5.72)	14.40 (5.01)	14.43 (5.13)
Aortic distensibility(10^−3^ mmHg^-1^)	2.04 (1.28)	1.86 (1.17)	2.12 (1.46)	2.16 (1.20)
Carotid YEM (mmHg)	1526 (780)	1675 (757)	1477 (734)	1426 (831)
Carotid DC (10^−3^ mmHg^-1^)	3.2 (1.3)	2.9 (1.2)	3.3 (1.2)	3.3 (1.3)
Carotid artery IMT (cm)	0.84 (0.17)	0.84 (0.16)	0.81 (0.15)	0.85 (0.21)
Carotid artery LD (mm)	0.42 (0.31)	0.16 (0.06)	0.34 (0.06)	0.75 (0.31)

Abbreviations: IMT = intima-media thickness; YEM = Young’s Elastic Modulus; DC = Distensibility coefficient; LD = longitudinal displacement.

### Associations with Longitudinal Displacement

Baseline characteristics by tertile of LD are also shown in Table 1. The average beat to beat variability in LD was 0.03 (0.29) mm. In univariate models, Chinese participants had less LD (β = -0.11, p = 0.02) compared to White participants. Greater LD was associated with greater carotid IMT (β = 0.26, p = 0.004). Unlike traditional measures of arterial stiffness (YEM and DC), there were no statistically significant univariate associations between LD and age, systolic blood pressure, diastolic blood pressure, pulse pressure, diabetes mellitus, former or current tobacco use or hsCRP. LD was not correlated significantly with markers of carotid stiffness including YEM (r = 0.005, β = 0.00, p = 0.92) or DC (r = 0.03, β = 9.32, p = 0.45), aortic distensibility (r = 0.04, β = 0.009, p = 0.56), or measures of small (r = 0.02, β = 0.002, p = 0.67) or large artery (r = -0.01, β = -0.001, p = 0.81) elasticity. In a multivariable model comprising age, sex, race, heart rate, and carotid IMT, Chinese (β = -0.10, p = 0.04) race/ethnicity and carotid IMT (β = 0.304 p = 0.003) were independently associated with LD.

### Longitudinal Displacement and Future CHD and CVD Events

Over the mean 9.5 year follow up period there were 19 (4.9%) total CVD events; 14 (3.6%) of these were CHD events. Although the number of events was small, after adjusting for age, sex, and race/ethnicity, we observed a notable but non-statistically significant trends toward greater LD predicting CHD (HR 3.27, 95% CI 0.96–11.14, p = 0.058) and CVD (HR 2.13, 95% CI 0.62–7.33, p = 0.232) events.

### Reproducibility of LV and LD measurements

**Fig 2:**
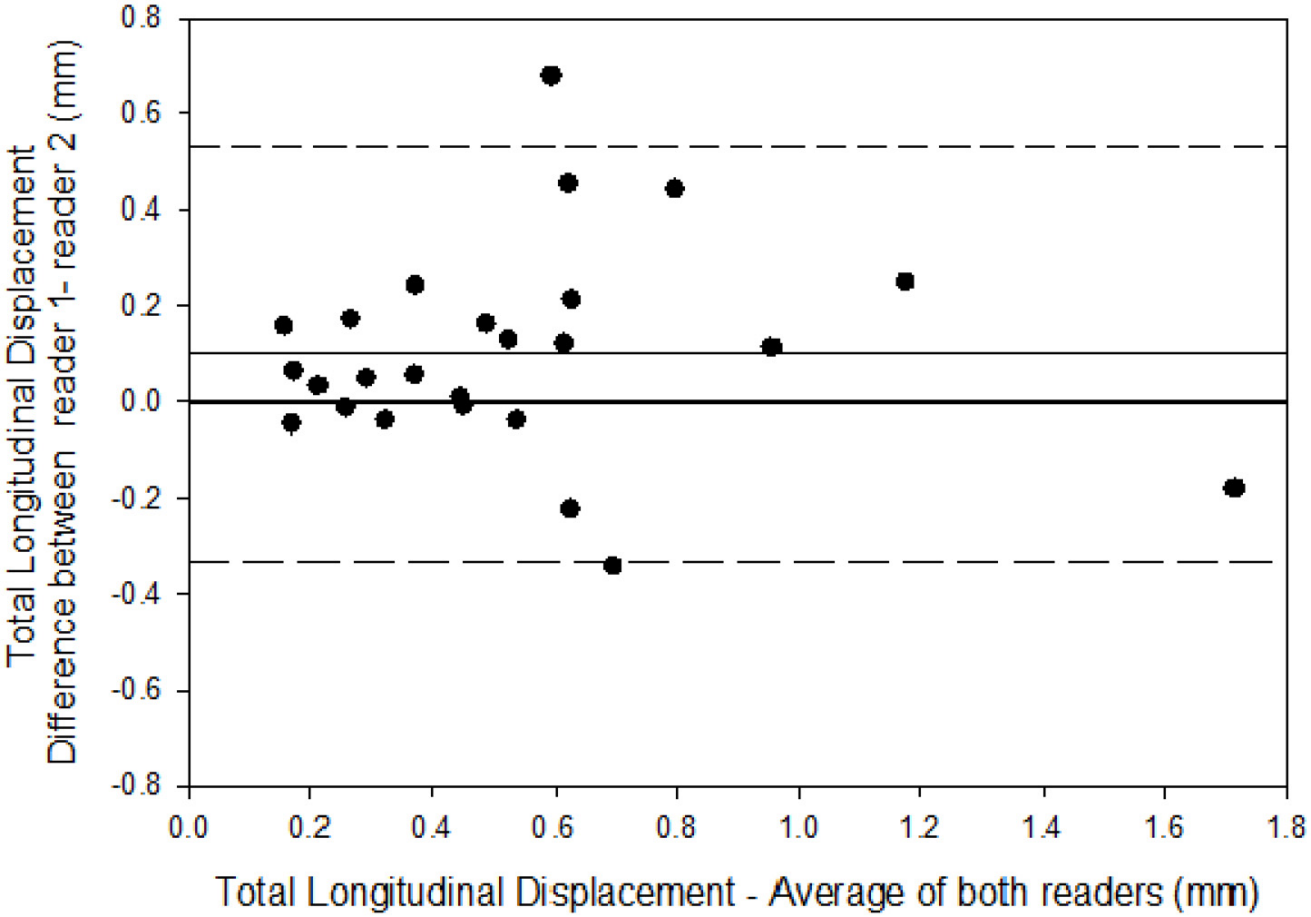
Bland-Altman Plot for Inter-Reader Reproducibility of Carotid Artery Longitudinal Displacement. The solid line at zero represents perfect inter-reader reproducibility. The second solid line at 0.012 represents the mean difference. The dashed lines represent +2 standard deviations (top) and -2 standard deviations (bottom) from the mean.

On comparison of 25 blinded replicates performed by two readers, there was a high correlation coefficient for total LD (r = 0.82) with mean (standard deviation) difference of 0.012 (0.093). Bland-Altman limits of agreement plots showed a small bias with rare outliers (1/25, Fig 2).

## Discussion

The most commonly obtained carotid arterial stiffness measurements typically evaluate radial or circumferential arterial displacement [2, 3] but they are cumbersome to obtain and only weakly predict future CVD events [24]. Furthermore, they assume that longitudinal arterial movement is negligible compared to radial movements and may be an inaccurate representation of arterial pathophysiology [4].

Prior studies that used VVI of the carotid artery to describe arterial motion were small [8, 9, 12], did not evaluate longitudinal measurements [13–15], made geometric assumptions about the carotid artery movement that may not be accurate [7, 8], and had divergent associations with CVD risk factors [7, 16, 17]. A standardized approach to measure carotid LD that was free of geometric assumptions and reproducible was developed. Using our approach, carotid artery LD was inversely associated with Chinese race/ethnicity and associated with carotid intima-media thickness. In MESA, Chinese individuals have lower CVD event rates and less carotid intima-media thickness and lower rates of IMT progression [19]. These observations provide internal consistency supporting our finding that increases in LD are associated with increased risk of CHD and CVD. This finding contradicts much [7, 17, 25] but not all [16] of the previously published literature. One study found that individuals with diabetes mellitus had significantly greater LD and pulse wave velocity compared to healthy controls [16]. Previous studies varied in their analytical software and tracking algorithms, points of interest, and the measurement protocols which likely impacted the results. The only other large study to evaluate LD of the carotid artery using VVI found the opposite association between LD and CVD events [7]. However, this study recruited high risk subjects with suspected CVD and used a software package designed for analysis of left ventricular motion [7]. The LD measurement software used geometric assumptions that may not be applicable to a tubular structure such as the carotid artery and included data points inside the vessel lumen, which could alter the true LD in the arterial wall [7]. Additionally, only one beat of the cardiac cycle was used in the prior study [7]. Although generally small, LD differences from beat to beat variation also could contribute to this discrepancy.

We believe that the technique used in the present study is an advance because it used a newer software version with a free trace feature that does not make any assumptions about vessel geometry and does not include information from speckles inside the vessel lumen. Additionally, LD was averaged over 2 cardiac cycles to account for a potential beat to beat variability

Of interest, however, no significant correlations between carotid artery LD and traditional measures of arterial stiffness such as carotid artery DC, carotid artery YEM, aortic distensibility, and either small or large artery elasticity were identified, nor was there a significant association with systolic blood pressure, challenging previous notions that LD truly is a measure of arterial stiffness [7, 8, 11, 16]. *In vitro* studies of passive mechanical properties of rat carotid arteries support our findings and suggest that with typical physiological deformation of the vessel, the longitudinal force and strain is nearly independent of internal pressure and the incremental elastic, but the radial incremental elastic moduli vary significantly with deformation [26].

In addition to representing superior movement or arterial translation, carotid artery LD may be affected by plaque presence, may be a marker of shear stress on the carotid wall or LD may be affected by differential movements between the arterial layers. Of note, the CCA typically is free of discreet atherosclerotic plaque and no plaques were seen in the CCA regions in this subset of the MESA participants. Changes in shear stress at the level of the endothelial layer could predispose the carotid wall to injury (causing higher carotid IMT, as observed) and increase the risk for CHD and CVD events. This is the first publication to attempt to describe LD in a large, ethnically diverse group, who are free of clinical CVD when studied.

Associations with measurements of LD and CVD risk factors have not been prospectively evaluated, especially in patients without known CVD. Prior to this study, there was no standardized protocol for measuring carotid artery longitudinal movements using ultrasound. It is clear, however, that the location of the region of interest within the vessel wall must be considered carefully. The cellular and molecular composition differ in each layer of the arterial wall, and the tunica media and adventitia layers of the artery may be tethered by deeper structures to a greater degree than the intima [9]. LD varies based on where the region of interest is placed in the wall. One study showed lower LD in patients with periodontal disease, however the region of interest was not uniform for every subject but rather at the midline of the intima and media border [17]. Therefore, subjects with thicker walls had a point of interest closer to the adventitia where more tethering likely resulted in decreased motion of the artery [17]. To avoid this pitfall, this study used points of interest that were uniformly chosen just under the intimal border to minimize IMT as a potential confounder. It is possible that intrinsic changes within the blood vessel wall would eventually lead to less LD in a more diseased artery, though differences between studies are more likely related to measurement technique.

### Limitations

These data are from a random subset of a large, well-established prospective longitudinal study, though the limitations of an observational study still apply. Image quality and reproducibility were excellent; however several factors did impact the LD measurements, including respiratory variation and transducer probe movement. The carotid ultrasound loops were long enough that these artifacts could be mitigated. However, both these artifacts and the absence of electrocardiographic gating could be a source of measurement error. Finally, the data regarding LD and CVD and CHD events are hypothesis-generating as there only were a small number of events in this subset and these analyses were not powered to detect changes in cardiovascular and coronary heart disease events. Further studies with additional events are needed to better characterize this finding.

## Conclusions

Greater carotid artery LD is with greater carotid wall thickness and less LD was associated with Chinese ethnicity. Greater LD may predict future CHD and CVD events. The conflicting results reported in the literature regarding the direction of LD and CVD risk may be attributed to differences in techniques.
